# Weather types and soil moisture modulate surface energy partitioning in a subtropical CAM pineapple field

**DOI:** 10.3389/fpls.2026.1835208

**Published:** 2026-05-11

**Authors:** Baoshan Zhao, Chengming Yan, Junjun He, Meian Dou, Zhijun Xu, Junbo Su, Dongsheng An

**Affiliations:** 1 South Subtropical Crops Research Institute, Chinese Academy of Tropical Agricultural Sciences/Key Laboratory of Tropical Fruit Biology, Ministry of Agriculture and Rural Affairs of China, Zhanjiang, China; 2 The United Graduate School of Agricultural Sciences, Ehime University, Matsuyama, Japan; 3 Guangdong Engineering Technology Research Center for Dryland and Water Saving Agriculture, Zhanjiang, China; 4 Zhanjiang Experimental Station, Chinese Academy of Tropical Agricultural Sciences, Zhanjiang, China

**Keywords:** Bowen ratio, crassulacean acid metabolism (CAM), energy flux, irrigation, pineapple, tropical fruit

## Abstract

Surface energy partitioning governs canopy thermal conditions and water use by determining how available energy is dissipated as latent versus sensible heat. In tropical and subtropical croplands, compound heat–drought events increase atmospheric evaporative demand, yet crassulacean acid metabolism (CAM) crops may respond differently from C_3_/C_4_ systems because daytime stomatal regulation constrains transpiration. Here we quantify subdaily energy partitioning in a subtropical CAM pineapple field in southern China using Bowen-ratio energy balance observations. Net radiation (*R_n_*), soil heat flux (*G*), and vertical gradients of temperature and vapor pressure were used to estimate sensible (*H*) and latent (*LE*) heat fluxes and to compute available energy (*A* = *R_n_*− *G*). To diagnose the coupling relationship between atmospheric demand and water supply, hourly data were classified into four weather types (WT4) using median thresholds of *R_n_* and vapor pressure deficit (VPD): LR_n_–LVPD (low *R_n_*, low VPD), LR_n_–HVPD (low *R_n_*, high VPD), HR_n_–LVPD (high *R_n_*, low VPD), and HR_n_–HVPD (high *R_n_*, high VPD). Soil water content (SWC) at a depth of 20 cm was further classified into three soil moisture states (SWC3) using percentiles (Dry, Normal, and Wet). Scenario medians and valid-hour counts were used to summarize LE/A, H/A, and G/Rn, complemented by Bowen ratio (*β = H/LE*) as an integrative indicator of sensible versus latent heat partitioning. The results showed that energy partitioning was dominated by compensation between *LE*/*A* and *H*/*A*, while *G*/*R_n_* remained small. *LE*/*A* was consistently lower under HR_n_–HVPD than under LR_n_–LVPD across soil-moisture states. Under LR_n_–LVPD, *LE*/*A* was 0.47 in Dry and Normal and increased to 0.56 in Wet; under HR_n_–HVPD it ranged from 0.26 (Dry) to 0.37 (Wet). Moisture effects were demand dependent: within HR_n_–HVPD, *LE*/*A* increased by 0.11 from Dry to Wet, whereas the LR_n_–LVPD Dry-to-Wet increase was 0.09. *β* responses reinforced this interaction. Under Dry conditions, *β* increased with VPD and remained high at high VPD, whereas under wet soils *β* was lower and tended to level off. Under HR_n_–HVPD, *β* declined steeply as SWC increased over 0.23–0.27 m^3^ m^-3^, then approached a plateau near 1.7; under non-HR_n_–HVPD conditions *β* showed weaker dependence and plateaued near 2.0. Composite daytime patterns further showed sustained *H* dominance under HR_n_–HVPD–Dry, while HR_n_–HVPD–Wet shifted partitioning toward higher *LE*. These results demonstrate that atmospheric demand sets a strong constraint on daytime evaporative cooling in this CAM pineapple system, and that soil water supply enhances latent heat dissipation most effectively under high-demand conditions, thereby informing model parameterization and targeted water management.

## 1 Introduction

The surface energy balance of croplands, particularly the partitioning of available energy into sensible and latent heat fluxes, constitutes a physical link between meteorological forcing, soil moisture, and plant physiological processes (Baldocchi, 2003; Lokupitiya et al., 2016; Zhou et al., 2025). In tropical and subtropical regions, croplands are increasingly exposed to compound heat–drought conditions (Li et al., 2017; Miralles et al., 2019). During such events, clear-sky radiation loads and atmospheric dryness rise concurrently, amplifying evaporative demand (Miralles et al., 2019; Tripathy et al., 2025). When soil moisture is limited and canopy conductance is constrained, available energy shifts toward sensible heat, increasing canopy warming and heat stress risk under joint atmospheric demand and soil moisture limitation (Berg et al., 2016; Xu et al., 2023; Chen et al., 2026). Because these partitioning shifts develop on subdaily timescales, their magnitude and duration can strongly shape crop exposure to heat extremes and transient water deficits during critical phenological stages (Teuling et al., 2010; Jaeger and Seneviratne, 2011). Quantifying how energy partitioning responds to meteorological forcing and soil moisture is therefore essential for understanding heat-risk emergence, guiding water-management strategies, and improving land-surface model parameterizations (Haghighi et al., 2018).

Pineapple (*Ananas comosus* (L.) Merr.) is a major cash crop in tropical and subtropical regions and is commonly grown under rainfed management (Hanyabui et al., 2024; Qiu et al., 2025). Unlike most C_3_ or C_4_ crops, pineapple exhibits crassulacean acid metabolism (CAM) photosynthesis (de Azevedo et al., 2007; Carr, 2012; Coelho et al., 2024; Liu et al., 2024). CAM shows a distinct diel pattern in gas exchange, with stomata typically opening at night for CO_2_ uptake and remaining relatively closed during daytime to reduce water loss (Hultine et al., 2019; Burgos et al., 2022). As a result, daytime latent heat flux may not increase proportionally with higher radiative forcing or atmospheric evaporative demand because stomatal regulation constrains canopy conductance and transpiration (Borland et al., 2014; Grossiord et al., 2020; Hultine et al., 2019). Under high vapor pressure deficit (VPD), stomatal closure can further suppress evapotranspiration, making evaporative limitation and enhanced sensible heating most likely during hot, dry periods when evaporative cooling would otherwise be most effective (Tuzet et al., 2003; Medlyn et al., 2011; Novick et al., 2016). This CAM-specific mismatch between atmospheric demand and evaporative response may modify surface temperature and land–atmosphere exchange, thereby amplifying compound heat and water stress under rainfed management (Berg et al., 2016; Miralles et al., 2019).

Many studies have examined pineapple water use, irrigation requirements, and seasonal ecohydrological dynamics, but important uncertainties remain at subdaily timescales (San-José et al., 2007; de Azevedo et al., 2007; Carr, 2012; Coelho et al., 2024). Solar radiation, VPD, and soil moisture can vary rapidly within a day, and energy partitioning often shows nonlinear, state-dependent responses. Analyses based on daily-to-seasonal aggregation may therefore mask short-term transitions and critical thresholds that govern the onset of evaporative limitation (Haghighi et al., 2018; Fu et al., 2024). However, atmospheric demand and soil water supply are often treated separately, and integrated frameworks that explicitly represent their interaction in governing the partitioning between latent and sensible heat remain scarce for rainfed CAM croplands (Haghighi et al., 2018; Miralles et al., 2019). This gap limits comparability across studies and constrains the direct application of observational evidence to management and model development.

This study addresses these gaps using observations from a typical subtropical pineapple field in southern China. Across coupled gradients of atmospheric demand and soil moisture, we examine how surface energy partitioning varies and when evaporative limitation emerges. Specifically, we aim to (1) characterize subdaily variability in latent and sensible heat partitioning across environmental conditions, (2) quantify the relative and interactive effects of meteorological forcing and soil moisture on energy allocation, and (3) identify critical conditions and sensitive periods associated with evaporative limitation. By framing energy partitioning as a conditional response to coupled demand–supply states, our results provide process-based constraints for land-surface model parameterizations and actionable insights for improving water use in pineapple production.

## 2 Materials and methods

### 2.1 Study site and experimental design

This study was conducted at the pineapple experimental station of the South Subtropical Crops Research Institute, Chinese Academy of Tropical Agricultural Sciences, in Zhanjiang, Guangdong Province, southern China (21°09′N, 110°16′E). Zhanjiang is a major pineapple-producing region and has a south-subtropical monsoon climate, with a mean annual air temperature of 23.6 °C, mean annual precipitation of 1619.6 mm, reference evapotranspiration (*ET*_0_) of 1242.7 mm, and annual sunshine hours of 1946.5 h (Zhao et al., 2023).

The experimental field covered 7,200 m^2^ (120 m × 60 m) and was surrounded predominantly by extensive pineapple plantations (>20,000 m²), ensuring a relatively homogeneous fetch for flux measurements. The cultivar was “Golden pineapple”, transplanted in March 2022 at a spacing of 1.2 m × 0.5 m. The soil is a lateritic red soil, with a mean bulk density of 1.23 g cm^-3^ in the 0–90 cm layer. Following local management practices, limited supplemental irrigation was applied only during the early growth stage to ensure establishment; thereafter, the system was primarily rainfed. Based on field management records and phenological observations, the growing season was divided into three distinct stages: a vegetative stage (1 April–31 December 2022), a flower induction/visible red stage (1 January–10 February 2023), and a fruit maturation stage (11 February–30 June 2023).

### 2.2 Instrumentation and data acquisition

**Figure 1:**
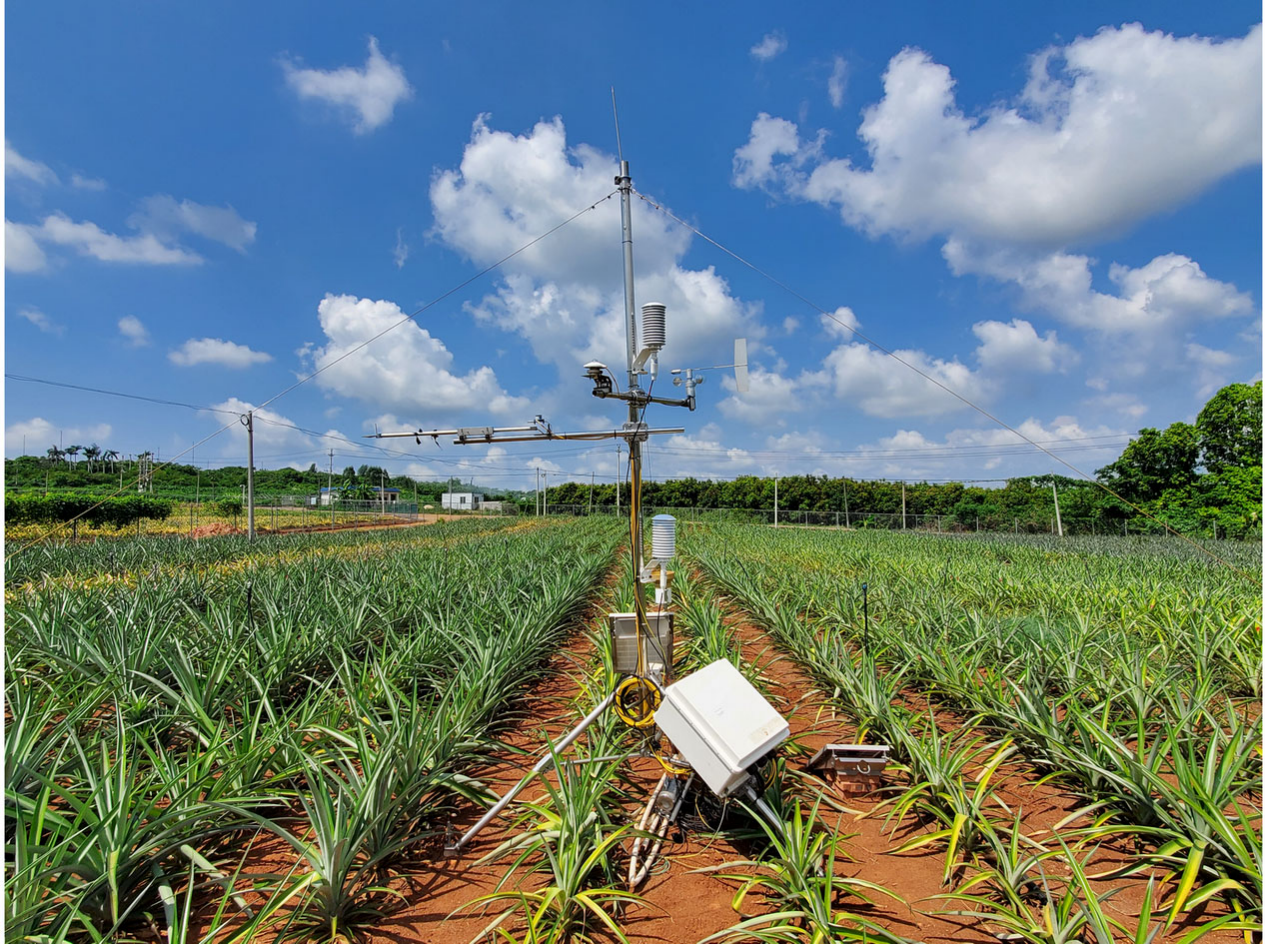
Layout of the Bowen–ratio energy balance observation system in the pineapple field.

Surface energy fluxes were measured using a Bowen-ratio energy balance (BREB) system, which combines net radiation and soil heat flux measurements with vertical gradients of air temperature and water vapor pressure to estimate sensible (*H*) and latent heat fluxes (*LE*) (**Figure 1**). Because southeasterly winds prevail year-round in the study area, the BREB system was installed in the southwestern part of the field to maximize upwind fetch. Under these conditions, the measured fluxes were considered representative of the pineapple canopy.

Net radiation (*R_n_*) was measured at 2.5 m above ground level using a CNR4 net radiometer (Kipp & Zonen, The Netherlands). Soil heat flux (*G*) was measured using two HFP01 heat flux plates (Hukseflux, The Netherlands) installed at approximately 2 cm below the soil surface, placed in representative interspaces (between rows and between plants within rows) and averaged to obtain *G*. Air temperature and relative humidity were measured at 1.5 m and 2.5 m using two HMP155A probes (Vaisala, Finland) to derive vertical gradients of temperature and vapor pressure for Bowen-ratio calculations. Wind speed and wind direction at 2.5 m were measured using a 03002 anemometer (R. M. Young, USA). Volumetric soil water content and soil temperature were measured using CS655 sensors (Campbell Scientific, USA) installed at depths of 5, 20, and 50 cm. Soil water content at 20 cm was used as an operational indicator of root-zone water supply, because pineapple has a shallow root system that generally extends to about 15–30 cm depth, placing 20 cm within the main rooting zone (Paull and Duarte, 2025).

All sensors were checked and calibrated prior to deployment. To minimize systematic errors in gradient measurements, the two temperature–humidity probes were co-located at the same height (2.0 m) before the experiment to verify consistency. All variables were sampled every 10 s and stored as 10 min averages using a CR3000 datalogger (Campbell Scientific, USA). Valid observations covered 12 July 2022 to 19 May 2023. Continuous micrometeorological measurements were collected during this period and were subsequently screened and aggregated for hourly and scenario-based analyses. For hourly analyses, quality-controlled 30 min records within the same hour were aggregated by averaging the two half-hourly values; hours with fewer than two valid half-hour records were treated as missing. After quality control and hourly aggregation, the dataset used for daytime scenario classification comprised 870 valid observations.

### 2.3 Energy flux calculations and quality control

The BREB method was used to estimate the sensible (*H*) and latent heat fluxes (*LE*) over the pineapple field using Equations 1–4. The Bowen ratio (*β*), defined as the ratio of *H* to *LE*, was calculated from the vertical gradients of air temperature and vapor pressure between the two measurement heights as follows (Bowen, 1926; Tanner et al., 1987):

(1) $$\beta =\frac{H}{LE}=\gamma \frac{\Delta T}{\Delta e}$$

where γ is the psychrometric constant (kPa K^-^¹), and Δ*T* (K) and Δ*e* (kPa) are the vertical gradients of air temperature and vapor pressure between the two measurement heights (Wu et al., 2024).

Available energy was defined as:

(2) $$A=R_{n}-G$$

where *R_n_* is net radiation (W m^-2^) and *G* is soil heat flux (W m^-2^). Fluxes were then calculated as:

(3) $$H=\frac{\beta A}{1+\beta }$$

(4) $$LE=\frac{A}{1+\beta }$$

Quality control followed the BREB-specific sensitivity to small gradients and sign inconsistencies, with particular attention to sunrise/sunset periods when numerical amplification may occur. Data were first screened using the sign-consistency rules recommended by Perez et al. (1999) (**Table 1**), based on *R_n_*-*G*, *Δe*, *β*, *H*, and *LE*. In addition, physically implausible values were removed using variable-specific ranges; for example, *LE* values outside -50 to 700 W m^-2^ were treated as outliers and excluded. To reduce instability associated with weak gradients, subsequent modelling and statistical analyses focused on daytime samples with positive and sufficiently large available energy. The exact threshold is reported with site-specific details. For analyses involving *β* periods with *LE* ≤ 0 or very small |*LE*| were excluded to avoid divergence.

**Table 1:** Sign-consistency criteria used to screen BREB data, following Perez et al. (1999).

Available energy	Vapor pressure gradient	Bowen ratio	Heat fluxes
*A*>0	*Δe*>0	*β*>–1	*LE*>0 and *H* ≤ 0 for −1< *β*≤ 0 or *H* > 0 for *β* > 0
	*Δe*<0	*β*>–1	*LE*<0 and *H* > 0
*A*<0	*Δe*>0	*β*>–1	*LE*>0 and *H* < 0
	*Δe*<0	*β*>–1	*LE*<0 and *H* ≥ 0 for −1< *β*≤ 0 or *H* < 0 for *β* > 0

### 2.4 Scenario classification and statistical analyses

To examine how atmospheric demand and soil water supply jointly control energy partitioning at the hourly scale, quality-controlled 10 min data were aggregated to hourly values of *R_n_*, *VPD*, *SWC*, *H*, *LE*, and *G*. Available energy was calculated by Equation 2, and energy partitioning was quantified as *LE*/*A*, *H*/*A*, and *G*/*R_n_*. Scenario classification and subsequent statistical analyses were restricted to daytime hours with *A* > 0 to avoid inflation of partitioning ratios under low-energy conditions. In addition, *G* was represented by direct measurements from the soil heat flux plates, without correction for soil heat storage above the plates, which may have slightly overestimated available energy. However, because the same instrumentation and processing were applied across all scenarios and *G* accounted for only a small fraction of the energy balance, the relative comparisons among scenarios are still valid.

Scenario categories were defined using quantile thresholds. The weather type classification (WT4) was derived by splitting hourly *R_n_* and VPD at their respective medians (P50) into “low” and “high” levels, which were then combined to form four classes: LR_n_–LVPD (low *R_n_*, low VPD), LR_n_–HVPD (low *R_n_*, high VPD), HR_n_–LVPD (high *R_n_*, low VPD), and HR_n_–HVPD (high *R_n_*, high VPD). Soil moisture states (SWC3) were defined using the 33rd and 66th percentiles (P33 and P66) of SWC to classify hourly conditions as Dry, Normal, or Wet. WT4 and SWC3 were defined as relative, site-specific environmental classes based on quantile thresholds of the observed hourly distributions. They were used to provide a reproducible demand–supply framework for comparing energy partitioning across contrasting conditions, rather than to define absolute physiological stress thresholds.

To characterize the nonlinear response of the Bowen ratio to environmental factors, a robust *β* was defined as the ratio of the median *H* to the median *LE*. Binned response curves were then constructed to examine *β* as a function of VPD under different SWC3 states and as a function of SWC under contrasting weather backgrounds (HR_n_–HVPD vs. Other). Equal-frequency binning was used to compute binned medians, uncertainty was quantified using bootstrap resampling to derive confidence bands, and kernel smoothing was applied for visualization where appropriate to highlight the overall nonlinear trends.

## 3 Results and analysis

### 3.1 Classification of weather types and soil moisture states

**Figure 2:**
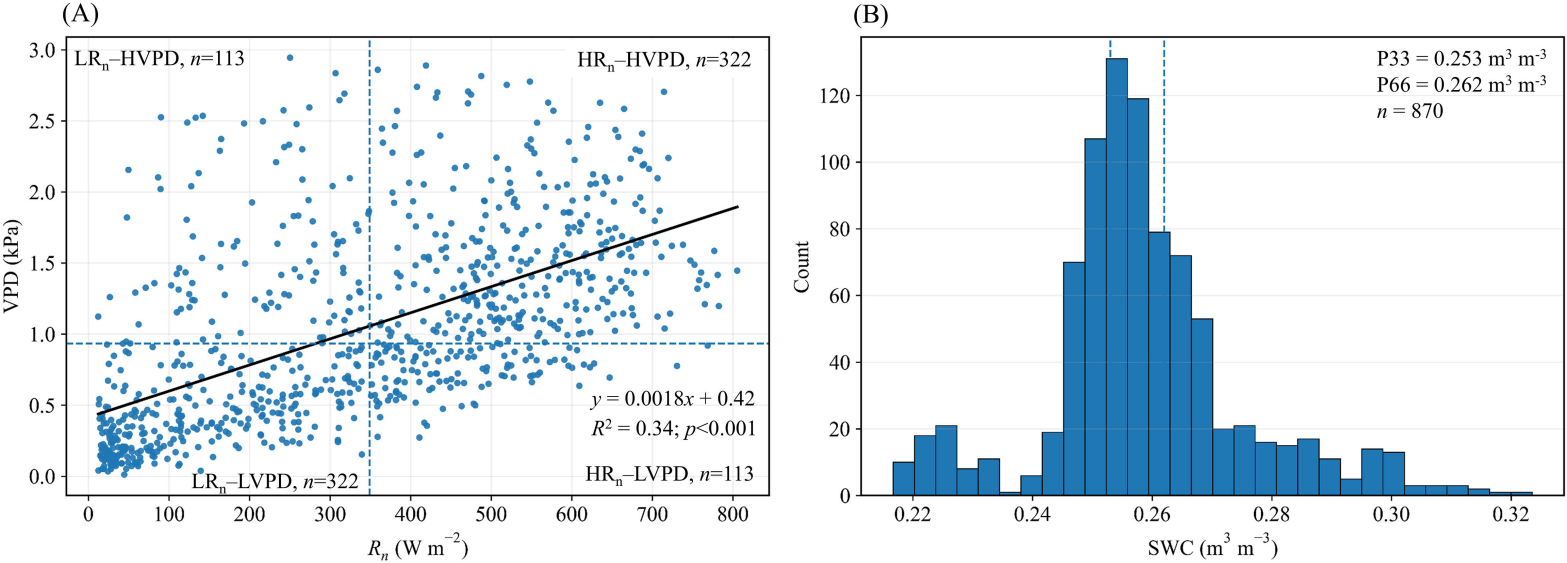
Definition and sample coverage of weather types and soil moisture (SWC) states. **(A)** Scatter of hourly *R_n_* versus VPD with P50 thresholds defining four weather types (LR_n_–LVPD, LR_n_–HVPD, HR_n_–LVPD, HR_n_–HVPD). **(B)** Histogram of hourly SWC with P33/P66 thresholds defining three moisture states (Dry, Normal, Wet).

**Figure 2** summarizes the classification framework for weather types and soil moisture states, defining the thresholds for subsequent scenario-based analyses. In **Figure 2A**, the bivariate distribution of hourly *R_n_* and VPD was partitioned using distribution-based median thresholds (P50; *R_n_* = 349 W m^−2^, VPD = 0.93 kPa), which defines four weather types (WT4): LR_n_–LVPD, LR_n_–HVPD, HR_n_–LVPD, and HR_n_–HVPD. The scatter pattern is wedge-shaped, with a wider VPD range under high–*R_n_* conditions and most high–VPD points occurring when *R_n_* is high, indicating systematic co-variation between radiation and atmospheric dryness at the hourly scale. A linear regression showed a significant positive relationship between *R_n_* and VPD (*R*^2^ = 0.336, *p* < 0.001), despite substantial scatter. Sample counts were uneven across categories (LR_n_–LVPD and HR_n_–HVPD: n=322 each; LR_n_–HVPD and HR_n_–LVPD: n=113 each), showing that LR_n_–LVPD and HR_n_–HVPD conditions were more frequent than the cross-category combinations, interpretations for LR_n_–HVPD and HR_n_–LVPD therefore warrant greater attention to uncertainty.

**Figure 2B** shows the hourly distribution of SWC and the thresholds used to define soil-moisture states. The 33rd and 66th percentiles (P33 = 0.253 and P66 = 0.262 m^3^ m^−3^; n=870) were used to classify soil moisture into three SWC states (SWC3): Dry, Normal, and Wet. SWC values clustered within 0.25–0.27 m^3^ m^−3^ and the P33–P66 range was narrow (0.009 m^3^ m^−3^), indicating limited variability during the study period. An extended upper tail up to 0.32 m^3^ m^−3^ suggests episodic wetting associated with rainfall, which provides Wet-state samples for comparison against Dry/Normal conditions. Given the narrow SWC range, the labels Dry, Normal, and Wet should be interpreted as relative moisture states within this field and observation period, rather than as definitive physiological drought classes. This classification links atmospheric demand (*R_n_* and VPD) with water supply (SWC) and provides the basis for the subsequent scenario-based energy-partitioning analyses.

### 3.2 Energy partitioning under different combinations of weather and soil moisture

**Figure 3:**
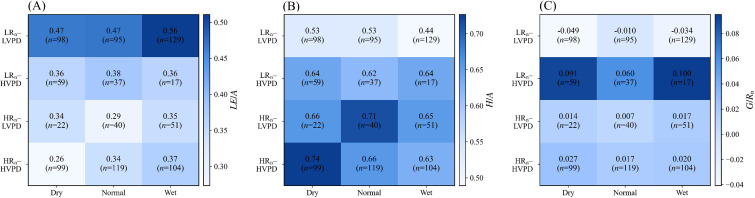
Scenario-based matrix of energy partitioning across weather types and soil-moisture states. Cells show medians with valid hourly counts (*n*), metrics are **(A)** *LE*/*A*, **(B)** *H*/*A*, and **(C)** *G*/*R_n_*.

To visualize the joint effects of weather types and SWC on energy partitioning, we constructed a scenario matrix based on WT4 × SWC3 (**Figure 3**). Cell values are medians with valid-hour counts (*n*). Across scenarios, energy partitioning variability is dominated by the compensation between *LE*/*A* and *H*/*A*, while *G*/*R_n_* remains comparatively small.

*LE*/*A* varies mainly with weather conditions, with soil moisture effects strongest under high atmospheric demand. Under LR_n_–LVPD, *LE*/*A* is 0.47 for both Dry (*n* = 98) and Normal (*n* = 95) and increases to 0.56 under Wet (n=129). Under HR_n_–HVPD, *LE*/*A* is consistently lower (0.26–0.37; *n* = 99, 119, and 104 for Dry, Normal, and Wet, respectively). The contrast in *LE*/*A* between LR_n_–LVPD and HR_n_–HVPD remained large, from −0.21 (45%) under Dry to −0.19 (34%) under Wet, indicating reduced latent heat allocation when high radiation coincides with high VPD, consistent with CAM-specific stomatal regulation under high evaporative demand. SWC effects on *LE*/*A* are strongly scenario dependent and are amplified under HR_n_–HVPD. Within HR_n_–HVPD, *LE*/*A* increases by +0.11 (42%) from Dry to Wet, while the Normal-to-Wet increment is smaller (+0.03), indicating reduced sensitivity at intermediate moisture. Under LR_n_–LVPD, the Dry-to-Wet increase is +0.09 (19%), showing a weaker moisture response than under HR_n_–HVPD. Cells with small sample sizes (e.g., LR_n_–HVPD–Wet, *n* = 17; HR_n_–LVPD–Dry, *n* = 22) display non-monotonic patterns and are not discussed further.

*H*/*A* shows the inverse pattern to *LE*/A, consistent with a redistribution between turbulent fluxes. Under LR_n_–LVPD, *H*/*A* decreases from 0.53 (Dry/Normal) to 0.44 (Wet), whereas under HR_n_–HVPD it decreases from 0.74 (Dry) to 0.63 (Wet). The HR_n_–HVPD Dry-to-Wet decrease in *H*/*A* (−0.11) matches the increase in *LE*/*A* (+0.11), suggesting that moisture-driven changes mainly reflect a shift between H and LE rather than changes in available energy. *G*/*R_n_* remained small across scenarios but changed sign under some conditions. Negative values under LR_n_–LVPD indicate upward release of previously stored soil heat under weak or declining radiation, due to reversal of the soil–surface temperature gradient. **Figure 3** indicates that HR_n_–HVPD–Dry is the most evaporatively limited state (lowest *LE*/*A* and highest *H*/*A*), while wetting under HR_n_–HVPD increases *LE*/*A* and reduces *H*/*A*, illustrating the combined control of atmospheric demand and soil water supply.

### 3.3 Relative contributions of weather type and soil moisture to latent heat fraction

**Figure 4:**
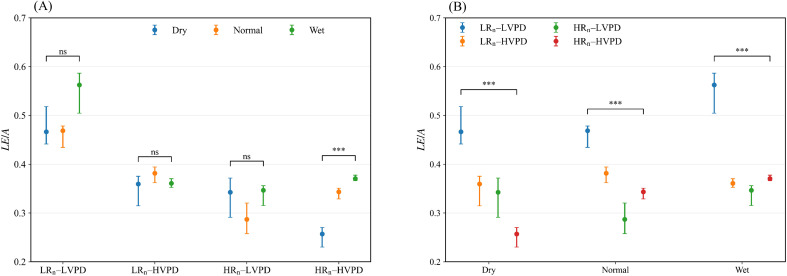
Comparisons of latent heat fraction (*LE*/*A*) under fixed weather types or fixed soil-moisture states. **(A)** SWC3 effects within each WT4; **(B)** WT4 effects within each SWC3. Points are medians with bootstrap confidence intervals; ns denotes non-significance, and *** denotes *p* < 0.001.

To separate the effects of atmospheric conditions and soil moisture on the latent heat fraction, we compared *LE*/*A* among SWC within each weather type and, conversely, among weather types within each SWC (**Figure 4**). Because some WT4 × SWC3 subsets had limited sample size or highly uneven numbers of valid hourly observations (e.g., LR_n_–HVPD–Wet and HR_n_–LVPD–Dry), **Figure 4** emphasizes the more robust contrasts, whereas low-count combinations were interpreted descriptively in **Figure 3** rather than highlighted in pairwise inference. SWC effects were clearly demand dependent (**Figure 4A**). Under HR_n_–HVPD, *LE*/*A* increased from 0.26 in the Dry state to 0.37 in the Wet state, an increase of 42%, whereas under LR_n_–LVPD the Dry-to-Wet increase was smaller (+0.09, 19%). Changes from Normal to Wet were comparatively small, suggesting reduced sensitivity around intermediate moisture levels. These contrasts are consistent with the scenario atlas (**Figure 3**), where moisture-driven shifts in partitioning were largest under high–demand conditions.

The influence of weather type remained pronounced across all soil moisture states. Under Dry conditions, *LE*/*A* decreased from 0.47 in LR_n_–LVPD to 0.26 in HR_n_–HVPD, a reduction of 44.7%. Under Normal conditions, *LE*/*A* declined from 0.47 to 0.34, corresponding to a 27.7% decrease. Under Wet conditions, *LE*/*A* decreased from 0.56 to 0.37, a reduction of 33.9%. All contrasts were significant (**Figure 4B**). These results show that HR_n_–HVPD conditions consistently reduced the *LE*/*A* even when soils were relatively wet, indicating strong atmospheric constraints on daytime evaporative cooling. Together, HR_n_–HVPD–Dry exhibited the lowest *LE*/*A*, whereas wetting under HR_n_–HVPD partly alleviated the reduction in *LE*/*A*.

### 3.4 Bowen ratio responses to atmospheric demand and soil moisture

**Figure 5:**
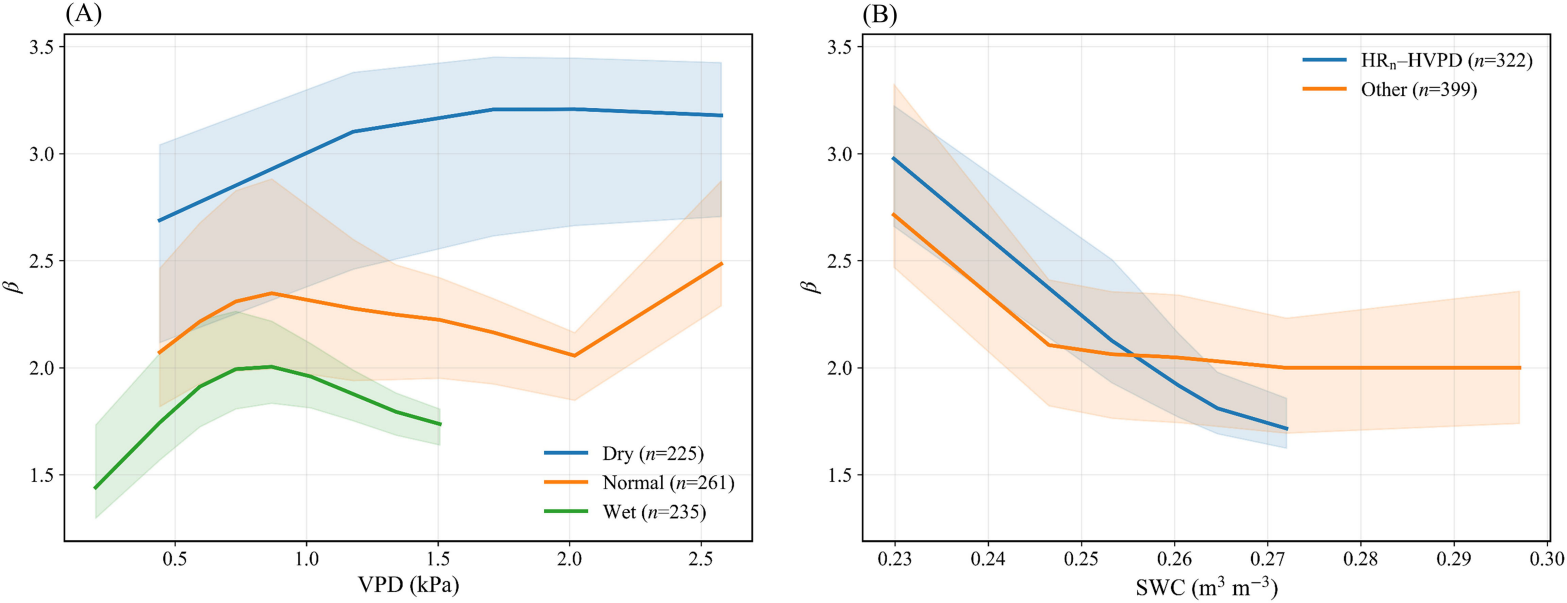
Bowen ratio (*β*) responses to atmospheric demand and soil moisture. **(A)** binned *β*–VPD curves stratified by SWC3; **(B)** binned *β*–SWC curves comparing HR_n_–HVPD versus Other. Solid lines show binned medians and ribbons denote bootstrap confidence bands.

The Bowen ratio (*β* = *H*/*LE*) provides a concise diagnostic of the relative importance of sensible versus latent heat dissipation. The *β*–VPD relationship showed distinct separation among soil moisture states (**Figure 5A**). Under Dry conditions (*n* = 225), *β* was highest and increased most strongly with VPD, reaching 2.7–3.0 at intermediate VPD (0.5–1.0 kPa) and remaining high at 3.1–3.2 under high VPD. Under Wet conditions (*n* = 235), *β* was lower at 1.4–2.0 and tended to level off after a modest peak at intermediate VPD. The Normal state (*n* = 261) was intermediate at 2.0–2.5 and showed a renewed increase at high VPD. These patterns are consistent with **Figures 3**, **4**, where Dry conditions were associated with lower *LE*/*A* and higher *H*/*A*, indicating that energy partitioning was more sensitive to atmospheric demand under lower-SWC conditions within the observed range.

The *β* response to soil moisture further depended on meteorological background (**Figure 5B**). Under HR_n_–HVPD conditions (*n* = 322), *β* declined strongly with increasing SWC: *β* was near 3.0 at low SWC, decreased most steeply over 0.23–0.27 m^3^ m^-3^, and then approached a plateau near 1.70 at higher SWC. Under non-HR_n_–HVPD conditions (*n* = 399), *β* showed a weaker dependence on SWC and reached a plateau near 2.0. This contrast matches the scenario atlas of **Figure 3**, where the Dry-to-Wet transition under HR_n_–HVPD produced the largest increase in *LE*/*A* and decrease in *H*/*A*. **Figure 5** translates the discrete scenario contrasts into continuous response functions: *β* remains high when high VPD coincides with dry soils, but declines rapidly with soil wetting under HR_n_–HVPD, supporting its use as an hourly-scale indicator of evaporative limitation and elevated heat-risk conditions.

### 3.5 Diurnal energy partitioning under representative scenarios

**Figure 6:**
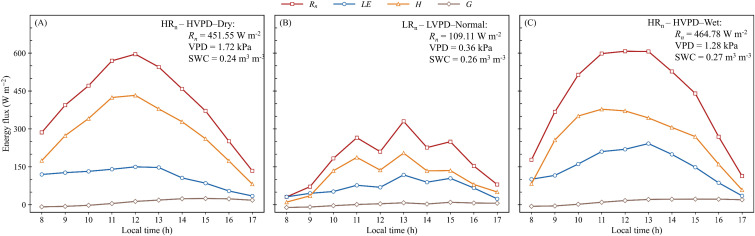
Composite hourly daytime energy fluxes under three selected scenarios. **(A)** HR_n_–HVPD–Dry, **(B)** LR_n_–LVPD–Normal, and **(C)** HR_n_–HVPD–Wet. Insets report mean *R_n_*, VPD, and SWC for 08:00–17:00 in each scenario.

To illustrate diurnal processes underlying the scenario-based statistics, **Figure 6** shows the composite hourly daytime patterns of *R_n_*, *LE*, *H*, and *G* under three selected scenarios. For each scenario, values at each hour from 08:00 to 17:00 were averaged across all valid days to characterize the typical daytime energy partitioning pattern. HR_n_–HVPD–Dry, LR_n_–LVPD–Normal, and HR_n_–HVPD–Wet were selected as representative scenarios because they clearly capture contrasts in atmospheric demand and soil-moisture background while also providing adequate sample coverage. Other WT4 × SWC3 combinations were not shown because they either occurred less frequently or were more affected by transitional or mixed forcing, resulting in less stable diurnal patterns.

Under HR_n_–HVPD–Dry conditions, mean daytime *R_n_*, VPD, and SWC were 451.55 W m^−2^, 1.72 kPa, and 0.24 m^3^ m^−3^, respectively. *R_n_* increased through the morning and peaked at about 570–600 W m^−2^ around midday. *H* rose rapidly with increasing radiation and remained higher than *LE* for most of the daytime period, reaching a broad midday maximum of 420–440 W m^−2^, whereas *LE* remained lower, at about 120–150 W m^−2^ from morning to early afternoon, and declined after 13:00. These patterns indicate strong evaporative limitation. Under LRn–LVPD–Normal conditions, mean daytime *R_n_*, VPD, and SWC were 109.11 W m^−2^, 0.36 kPa, and 0.26 m^3^ m^−3^, respectively. All fluxes were much smaller than under the HR_n_–HVPD scenarios. *R_n_* remained low, and both *H* and *LE* showed weak diurnal variation. The difference between *H* and *LE* was also smaller, indicating that low radiative input constrained the magnitude of turbulent energy exchange. Consistent with an energy-limited regime, *G* was near zero and varied little. Under HR_n_–HVPD–Wet conditions, mean daytime *R_n_* remained high at 464.78 W m^−2^, close to that under HR_n_–HVPD–Dry, whereas mean VPD was lower (1.28 kPa) and mean SWC was higher (0.27 m^3^ m^−3^). *R_n_* again peaked near 600 W m^−2^ at midday. Compared with HR_n_–HVPD–Dry, *LE* increased more strongly from late morning to early afternoon and reached about 200–250 W m^−2^, while the midday *H* peak was lower at about 370–390 W m^−2^. Thus, wetting shifted daytime partitioning toward *LE* and reduced sensible heating, although *H* remained the dominant turbulent flux.

## 4 Discussion

### 4.1 CAM constraints on latent heat partitioning under high atmospheric demand

A key result is that the latent heat fraction (*LE*/*A*) was lower under HR_n_–HVPD than under LR_n_–LVPD, and this contrast was observed across Dry, Normal, and Wet soil-moisture states. Even when soils were relatively wet, *LE*/*A* under HR_n_–HVPD remained below LR_n_–LVPD, indicating that higher atmospheric evaporative demand does not necessarily lead to a higher latent heat fraction.

This result is consistent with the diel gas-exchange strategy of CAM plants, which typically open stomata at night for CO_2_ uptake and remain relatively closed during daytime to limit water loss (Osmond, 1978; Lüttge, 1987; Hultine et al., 2019). Daytime stomatal regulation therefore constrains canopy conductance and transpiration, and this constraint tightens at elevated VPD. More generally, feed-forward stomatal responses to VPD reduce conductance as atmospheric dryness increases, limiting evaporative cooling and shifting available energy toward sensible heating (Oren et al., 1999; Tuzet et al., 2003; Medlyn et al., 2011; Grossiord et al., 2020).

Observations in pineapple systems support this interpretation. Field studies have reported energy partitioning distinct from many C3/C4 cropping systems and highlighted nonlinear responses of latent heat flux to environmental drivers (San-José et al., 2007; Liu et al., 2024). Work on pineapple water use and irrigation requirements also indicates that evapotranspiration is controlled by interacting meteorological and soil-moisture constraints (de Azevedo et al., 2007; Carr, 2012). Similar demand–response decoupling has been reported across ecosystems, where rising VPD reduces canopy conductance and weakens evaporative cooling (Novick et al., 2016; Grossiord et al., 2020). Ecosystem-scale studies of CAM vegetation further suggest joint limitation of transpiration by VPD and soil moisture and emphasize the day–night asymmetry of CAM water loss (Wohlfahrt et al., 2012; Kohonen et al., 2026). Together, these lines of evidence suggest that persistently low *LE*/*A* under HR_n_–HVPD reflects coupling between CAM physiological regulation and atmospheric dryness, alongside radiative forcing, rather than radiation alone (Borland et al., 2014; Hultine et al., 2019).

### 4.2 Interaction between atmospheric demand and soil moisture

A second key result is the clear context dependence of moisture effects on energy partitioning. Soil wetting was associated with the largest increase in *LE*/*A* under HR_n_–HVPD, and *β* showed its strongest decline with increasing SWC under HR_n_–HVPD, forming a moisture-sensitive window that transitioned to a plateau. This result is consistent with land–atmosphere coupling studies indicating that energy partitioning becomes more sensitive to soil moisture at higher radiation and VPD. Under Dry conditions, moisture sensitivity promotes a shift toward sensible heating, whereas under wetter conditions sensitivity weakens as the system moves toward an energy-limited regime (Seneviratne et al., 2010; Miralles et al., 2019). At regional to global scales, soil moisture–atmosphere feedbacks can amplify warming during droughts and heatwaves through reduced evaporative cooling (Teuling et al., 2010; Berg et al., 2016). From an evaporation-regime perspective, the transition between moisture-limited and energy-limited behavior depends on atmospheric demand, providing a conceptual basis for the moisture-sensitive window identified here (Haghighi et al., 2018; Vargas Zeppetello et al., 2019). Because SWC3 was defined from percentiles and the observed SWC range was narrow, Dry, Normal, and Wet should be interpreted as relative moisture states rather than physiological drought thresholds. In addition, part of the apparent SWC effect may reflect co-variation with radiation and atmospheric demand, with moisture sensitivity becoming more evident under high-demand conditions.

Field observations also show that evaporative fraction and *β* are controlled by interactions among VPD, *R_n_*, and soil water status, with moisture constraints becoming most evident under high-demand conditions (Crago and Brutsaert, 1996; Gu et al., 2006; Tong et al., 2022). In this study, the rapid decline of *β* with increasing SWC under HR_n_–HVPD followed by stabilization is consistent with a transition from strong moisture sensitivity to weaker sensitivity as soils become wetter (Haghighi et al., 2018; Vargas Zeppetello et al., 2019). Although SWC varied within a relatively narrow range during the study period, clear partitioning shifts emerged specifically under HR_n_–HVPD. This may reflect, first, the shallow effective rooting depth of pineapple, whereby modest changes in moisture within the active layer can lead to measurable changes in plant water availability and evapotranspiration (Carr, 2012; de Azevedo et al., 2007). Second, if daytime transpiration is physiologically constrained in CAM plants, the relative contribution of soil evaporation to daytime *LE* may increase, making near-surface water availability more influential under concurrent high radiation and high VPD. This interpretation is consistent with pineapple field observations reporting strong soil-moisture influences on *LE* and complex interactions with meteorological drivers (San-José et al., 2007; Liu et al., 2024). From a management perspective, evidence on pineapple water productivity and mulching/evaporation-reduction practices also highlights the value of maintaining effective root-zone water supply when marginal cooling benefits are greatest (Coelho et al., 2024; Carr, 2012).

HR_n_–HVPD–Dry represents an evaporative-limitation state, with the lowest *LE*/*A*, highest *H*/*A*, and highest *β*. Under HR_n_–HVPD, soil wetting increased *LE*/*A* and reduced *β*, indicating that improved water supply can reopen the latent heat pathway and shift partitioning away from sensible heating. This is consistent with the land–atmosphere coupling framework in which water availability becomes a key bottleneck under strong atmospheric demand and wetting yields a large enhancement of evaporative cooling (Seneviratne et al., 2010; Teuling et al., 2010; Miralles et al., 2019).

### 4.3 Implications for agroecosystem model parameterization and field management

A two-dimensional demand–supply classification based on *R_n_*–VPD and SWC, combined with continuous *β* responses, provides implications for land-surface and agroecosystem modelling. The results show that variation in the latent heat fraction cannot be captured by *R_n_* or SWC alone: the weather type was associated with consistently lower or higher *LE*/*A* across moisture states, while SWC effects on *LE*/*A* were strongest under HR_n_–HVPD. This points to the need for evapotranspiration and energy-partitioning schemes to include explicit demand–supply interactions, rather than prescribing soil-moisture sensitivity as invariant across meteorological conditions. Without such interactions, models may underestimate the shift from latent to sensible heat during dry, high-demand periods and the associated amplification of heat extremes (Tuzet et al., 2003; Medlyn et al., 2011; Novick et al., 2016; Berg et al., 2016). For CAM systems in particular, representing stomatal regulation under high VPD and its coupling with water limitation is essential for reproducing daytime evaporative constraints (Oren et al., 1999; Borland et al., 2014; Grossiord et al., 2020).

From a management perspective, the highest heat-risk conditions occur under HR_n_–HVPD–Dry, when evaporative cooling is most limited and sensible heating dominates. Under HR_n_–HVPD, wetter root-zone conditions increase *LE*/*A* and lower *β*, indicating greater evaporative cooling potential. These results support a targeted-timing strategy for rainfed systems: rather than maintaining uniform soil moisture under all conditions, limited interventions—through conservation practices or supplemental irrigation—are likely to yield the greatest marginal benefit when applied prior to or during periods of concurrent high radiation and high VPD. This timing-based approach aligns with pineapple irrigation studies and evidence that improving effective root-zone supply can increase water productivity (de Azevedo et al., 2007; Carr, 2012; Coelho et al., 2024).

Two methodological issues merit attention for strengthening inference and generality. First, nocturnal stomatal opening and associated nighttime fluxes are a defining feature of CAM and may contribute to daily water and energy budgets; accounting for nighttime processes is therefore needed for a complete mechanistic picture of CAM surface exchange (Wohlfahrt et al., 2012; Whelan et al., 2018; Kohonen et al., 2026). Second, hourly-scale partitioning is sensitive to measurement uncertainty and filtering choices; energy-balance non-closure and methodological differences can affect the absolute magnitudes of *H* and *LE* and should be considered when comparing scenarios and interpreting effect sizes (Wilson et al., 2002; Foken, 2008). Overall, the results suggest a hierarchy of controls in subtropical CAM pineapple systems: atmospheric demand sets the forcing background, while soil water supply governs whether the latent heat pathway can be activated under high-demand conditions, providing a practical basis for identifying heat-risk windows and targeted mitigation (Miralles et al., 2019; Grossiord et al., 2020). Future work should integrate nighttime fluxes with plant physiological observations to better resolve diel CAM regulation and determine whether the relative soil-moisture classes identified here correspond to biologically meaningful thresholds.

## 5 Conclusion

This study quantified subdaily surface energy partitioning in a rainfed CAM pineapple field using Bowen-ratio energy balance observations and a demand–supply classification based on radiation, atmospheric dryness, and root-zone soil moisture. Across the observation period, variability in partitioning was dominated by compensation between latent and sensible heat fractions, whereas the ground heat flux term was relatively small and mainly reflected short-term heat storage.

First, high atmospheric demand conditions exerted a persistent constraint on the latent heat fraction. HR_n_–HVPD conditions consistently reduced *LE*/*A* relative to LR_n_–LVPD across Dry, Normal, and Wet states. Under LR_n_–LVPD, *LE*/*A* was 0.47 in both Dry and Normal and increased to 0.56 in Wet. Under HR_n_–HVPD, *LE*/*A* remained lower, ranging from 0.26 (Dry) to 0.37 (Wet). These results show that increased atmospheric evaporative demand does not necessarily yield a higher latent heat share in this CAM system; instead, daytime evaporative cooling remains constrained under HR_n_–HVPD, shifting partitioning toward sensible heating. The LR_n_–LVPD to HR_n_–HVPD reduction in *LE*/*A* remained large across moisture backgrounds, indicating a robust atmospheric control on daytime evaporative cooling potential.

Second, within the observed SWC range, relative soil-moisture effects were strongly demand dependent and were amplified under HR_n_–HVPD. Within HR_n_–HVPD, *LE*/*A* increased by 0.11 from Dry to Wet, whereas under LR_n_–LVPD the Dry-to-Wet increase was 0.09 and changes near intermediate moisture were limited. The inverse responses of *H*/*A* mirrored *LE*/*A*, indicating that soil moisture primarily redistributes turbulent fluxes between *LE* and *H* rather than altering available energy.

Third, Bowen ratio *β* responses provided continuous diagnostics of these controls. Under Dry conditions, *β* increased with VPD and remained high at the upper VPD range. Under HR_n_–HVPD, *β* declined steeply as SWC increased over 0.23–0.27 m^3^ m^-3^ and then approached a plateau near 1.7; under non-HR_n_–HVPD conditions, *β* showed weaker dependence and plateaued near 2.0. This identifies a moisture-sensitive window under high demand, within which wetting yields the largest reduction in β and the strongest shift away from sensible heating.

Composite daytime patterns supported these conclusions: HR_n_–HVPD–Dry showed sustained midday H dominance despite high radiation, whereas HR_n_–HVPD–Wet produced substantially higher *LE* that approached *H*; low-radiation conditions constrained all fluxes and muted partitioning contrasts. Together, the scenario atlas, effect-size contrasts, response curves, and diurnal patterns indicate a clear hierarchy of controls in CAM pineapple systems: atmospheric demand sets the background constraint on daytime evaporative cooling, while soil water supply governs the extent to which the latent heat pathway can be engaged under HR_n_–HVPD forcing.

Practically, HR_n_–HVPD–Dry represents the highest heat-risk window because it combines low *LE*/*A* with high *H*/*A*. Maintaining higher root-zone moisture during high-demand periods is therefore likely to provide the greatest cooling benefit, supporting targeted timing of conservation measures or supplemental irrigation. For modelling, the results support parameterizations that include demand × supply interactions and represent CAM-specific conductance constraints under high VPD. Future work should incorporate nighttime CAM fluxes and better quantify uncertainty from BREB screening and energy-balance non-closure when extending these conclusions across sites and seasons.

## Data Availability

The raw data supporting the conclusions of this article will be made available by the authors, without undue reservation.
